# Supplementary material for: Thermal cycling protects SH-SY5Y cells against hydrogen peroxide and β-amyloid-induced cell injury through stress response mechanisms involving Akt pathway
Source: PLoS One. 2020 Oct 1;15(10):e0240022. doi: 10.1371/journal.pone.0240022 (PMC7529293; doi:10.1371/journal.pone.0240022)
Supplement: S1 File — (PDF) [file pone.0240022.s001.pdf]

Fig 2A

H2O2 (uM)	0	150	200	250	300	350	400	450
	102.1739	87.6087	81.52174	83.91304	58.69565	63.04348	65.21739	52.17391
Normalized	100	87.17391	85.86957	82.6087	76.08696	73.91304	56.52174	52.17391
Viability	97.82609	80.86957	71.95652	66.52174	78.26087	78.26087	54.34783	54.34783
average	100	85.21739	79.78261	77.68116	71.01449	71.73913	58.6956533	52.89855
H2O2 (uM)	500							
	43.47826							
Normalized	40							
Viability	37.3913							
average	40.28985							

Fig 2B

	C		41.5 TC		41.5 HT	
H2O2 (uM)	0	450 uM	0	450 uM	0	450 uM
	97.84367	57.42049	102.965	74.89757	99.46092	62.27224
Normalized	101.0782	54.71698	101.3477	67.96765	100.8598	65.15903
Viability	101.0782	49.25606	99.31267	62.93261	95.01617	58.31536
average	100	53.79784	101.2084	68.59928	98.44564	61.9155433

Fig 2C

	C		42.5 TC x 8		42.5 HT 2hr		42.5 TC x 12	
H2O2 (uM)	0	450 uM	0	450 uM	0	450 uM	0	450 uM
	97.84367	57.42049	99.57746	79.5493	101.9718	62.67606	92.01481	78.21259
Normalized	101.0782	54.71698	102.6761	82.43662	95.38028	69.4169	94.49075	81.54416
Viability	101.0782	49.25606	100.2817	84.32394	101.1268	63.85915	92.96668	74.40508
average	100	53.79784	100.8451	82.10329	99.49296	65.31737	93.1574133	78.05394
	42.5 HT 3hr							
H2O2 (uM)	0	450 uM						
	75.61185	42.35854						
Normalized	72.13591	42.04125						
Viability	73.4532	40.29614						
average	73.73365	41.56531						

Fig 2D

	C	H2O2	1min	3min
	110.5	59.6	62.8	58.2
	98.2	63.5	58.1	64.5
Normalized	99.3	59.9	57.8	63.7
Viability	92.1	54.9	58.5	62.1
average	100.025	59.475	59.3	62.125

Fig 2E

	C		42.5 HT 2hr		42.5 TC x8	
	0	450 uM	0	450 uM	0	450 uM
	106.821	214.286	112.943	221.214	110.849	169.012
Normalized	92.803	218.153	101.987	218.475	103.598	142.75

Viability	100.376	217.508	103.598	188.507	96.831	144.844
average	100	216.649	106.176	209.3987	103.7593	152.202

Fig 3A

Abeta (uM)	0	25	50
	106.5903	63.03725	57.30659
Normalized	94.26934	61.60458	51.86246
Viability	99.1404	60.45845	54.44126
average	100	61.70009	54.53677

Fig 3B

	C	Abeta	TC (pre)	TC (post)	HT (pre)	HT (post)
	100.274	60.54314	65.47092	77.52475	59.29393	53.40594
Normalized	103.0137	55.12871	56.18326	82.49363	52.27496	59.06647
Viability	96.71233	58.32815	61.34478	84.21641	56.25922	55.62094
average	100	58	60.99965	81.4116	55.9427	56.0311167

Fig 4

	C	H2O2	TC+H2O2	HT+H2O2
ROS level	100	167.9845	129.4574	162.5323
normalized to	100	149.2647	121.0784	163.5784
control	100	155.2339	129.176	151.0022

Fig 5

	C	H2O2	TC+H2O2	HT+H2O2
	33.5	50.4	39	42
Cells with	35	51.9	35	45.9
decreased	39	52	34	41
MMP (%)	33.3	49.4	37.2	44.2

Fig 9A	C	H2O2	12LY+H	25LY+H	TC+H	TC+H+12LY	TC+H+25LY
	99.14984	47.50266	46.54623	46.54623	88.94793	62.16791	51.64718
Normalized	96.28055	66.95005	42.08289	38.57598	88.31031	68.86291	51.32837
Viability	104.5696	61.53029	52.60361	53.87885	79.70244	67.58767	49.09671
average	100	58.661	47.07758	46.33369	85.65356	66.2061633	50.6907533
