# Supplementary material for: Thermal cycling protects SH-SY5Y cells against hydrogen peroxide and β-amyloid-induced cell injury through stress response mechanisms involving Akt pathway
Source: PLoS One. 2020 Oct 1;15(10):e0240022. doi: 10.1371/journal.pone.0240022 (PMC7529293; doi:10.1371/journal.pone.0240022)
Supplement: S2 File — (PDF) [file pone.0240022.s002.pdf]

Fig 6

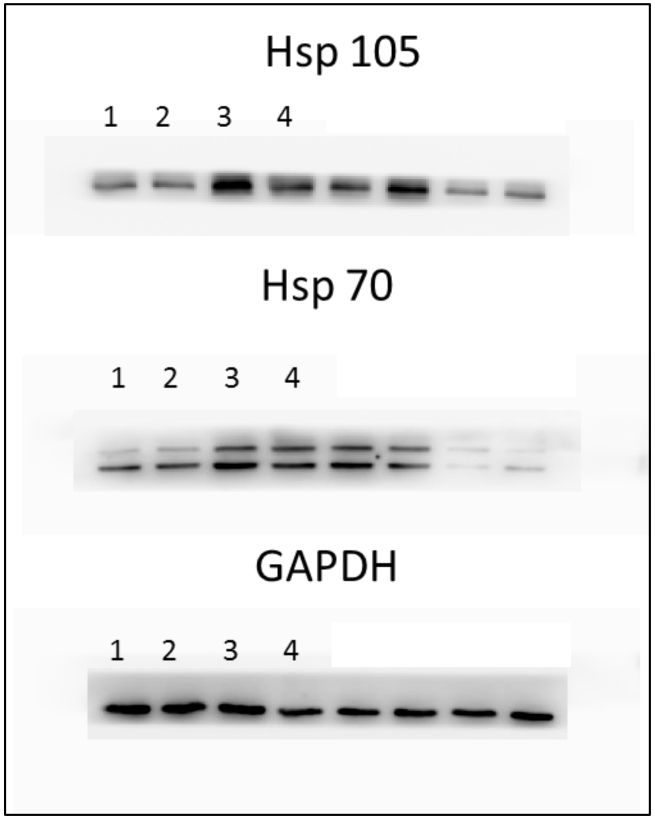

1: C

2: H_2O_2

3: TC+H_2O_2

4: HT+H_2O_2

Fig 7

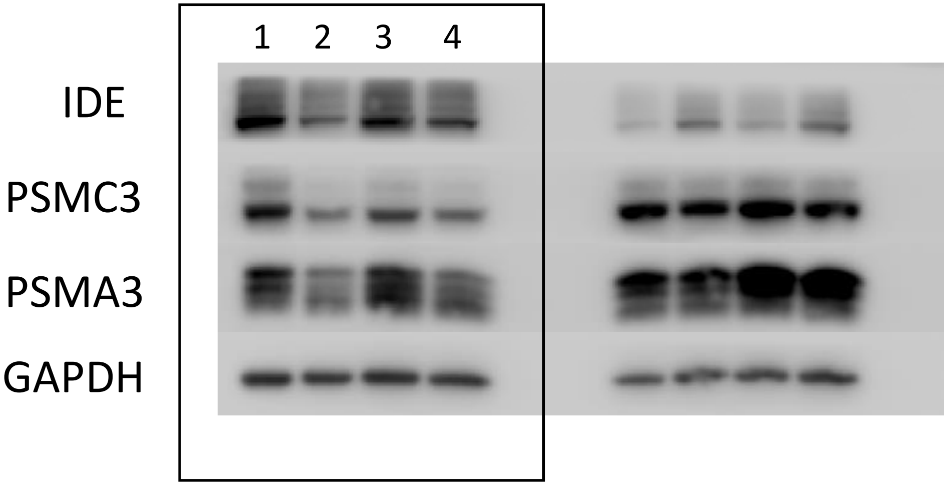

1: C

2: H_2O_2

3: TC+H_2O_2

4: HT+H_2O_2

Fig 8

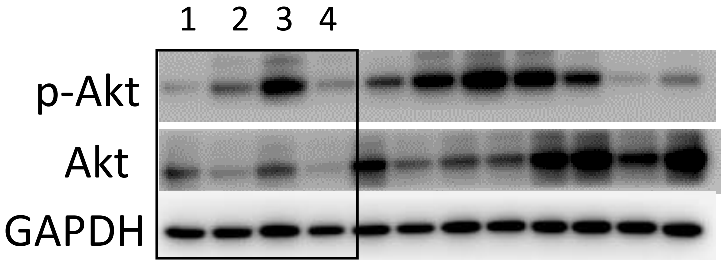

1: C

2: H_2O_2

3: TC+H_2O_2

4: HT+H_2O_2

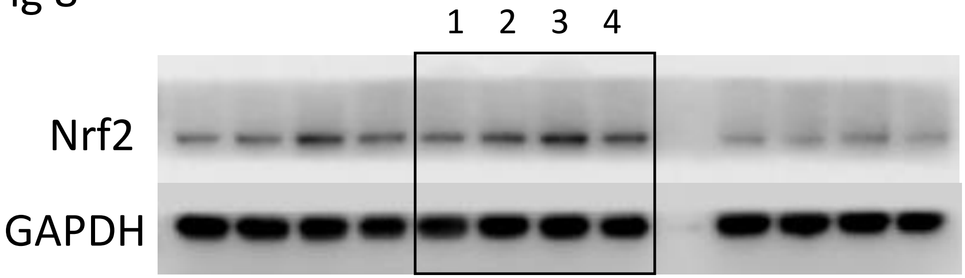

1: C

2: H_2O_2

3: TC+H_2O_2

4: HT+H_2O_2

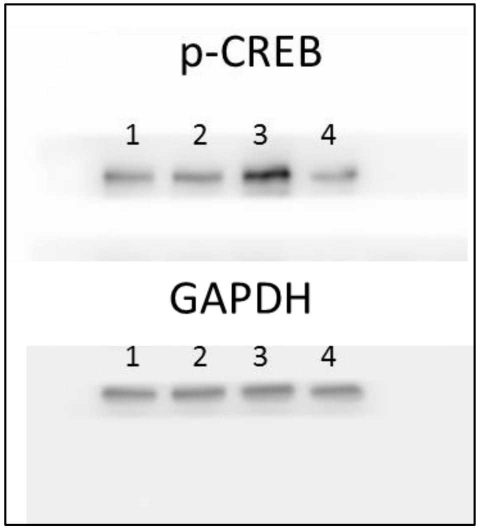

1: C

2: H_2O_2

3: TC+H_2O_2

4: HT+H_2O_2

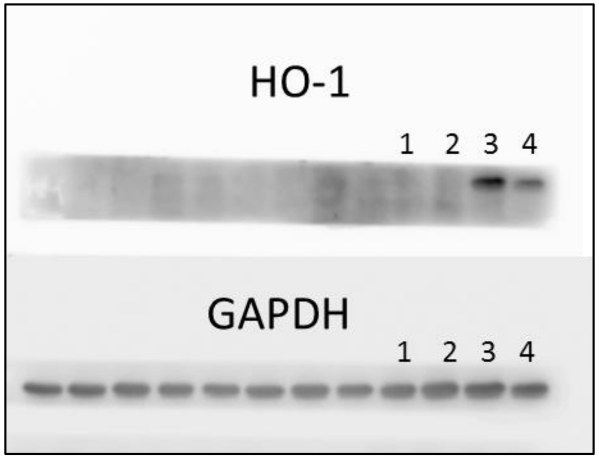

1: C

2: H_2O_2

3: TC+H_2O_2

4: HT+H_2O_2

Fig 9

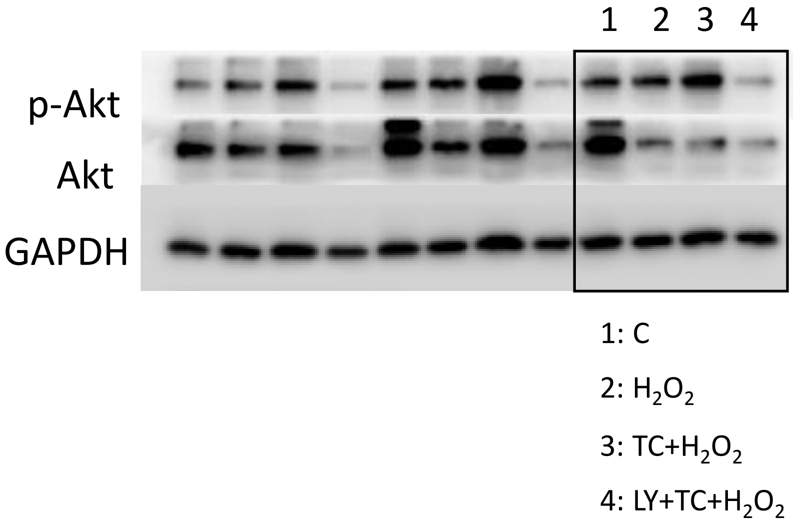
1 2 3 4
p-Akt
Akt
GAPDH
1: C
2: H_2O_2
3: TC+H_2O_2
4: LY+TC+H_2O_2

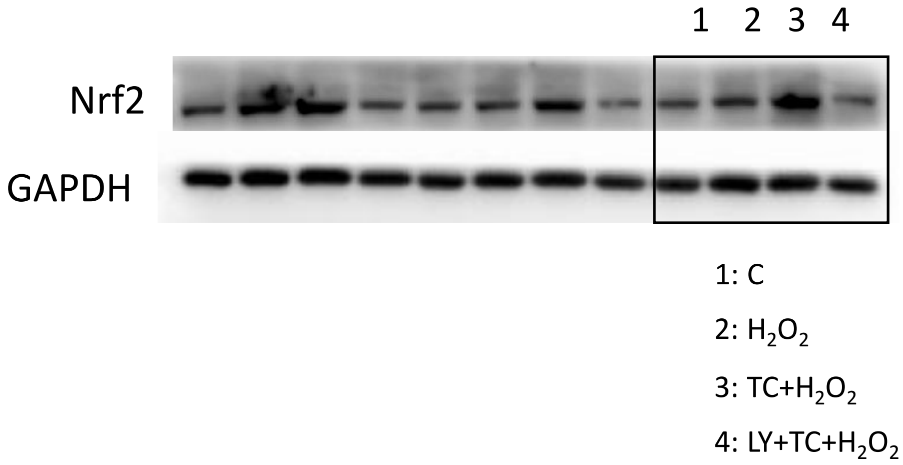
1 2 3 4
Nrf2
GAPDH
1: C
2: H_2O_2
3: TC+H_2O_2
4: LY+TC+H_2O_2
